# Modulating the Oxygen Reduction Reaction Performance via Precisely Tuned Reactive Sites in Porphyrin-Based Covalent Organic Frameworks

**DOI:** 10.3390/molecules28124680

**Published:** 2023-06-09

**Authors:** Xiaoqing Liang, Zhi Zhao, Ruili Shi, Liting Yang, Bin Zhao, Huijie Qiao, Lipeng Zhai

**Affiliations:** 1 School of Materials Science and Engineering, Taizhou University, Taizhou 318000, China; liangxq@tzc.edu.cn (X.L.); zhaobin007@tzc.edu.cn (B.Z.); 2 School of Mathematics and Physics, Hebei University of Engineering, Handan 056038, China; shiruili@hebeu.edu.cn; 3 Henan Key Laboratory of Functional Salt Materials, Center for Advanced Materials Research, Zhongyuan University of Technology, Zhengzhou 450007, China; 6702@zut.edu.cn

**Keywords:** covalent organic frameworks, oxygen reduction reaction, porous materials

## Abstract

Covalent organic frameworks (COFs) have emerged as promising electrocatalysts due to their controllable architectures, highly exposed molecular active sites, and ordered structures. In this study, a series of porphyrin-based COFs (TAPP-x-COF) with various transition metals (Co, Ni, Fe) were synthesized via a facile post-metallization strategy under solvothermal synthesis. The resulting porphyrin-based COFs showed oxygen reduction reaction (ORR) activity with a trend in Co > Fe > Ni. Among them, TAPP-Co-COF exhibited the best ORR activity (*E*_1/2_ = 0.66 V and jL = 4.82 mA cm^−2^) in alkaline media, which is comparable to those of Pt/C under the same conditions. Furthermore, TAPP-Co-COF was employed as a cathode in a Zn-air battery, demonstrating a high power density of 103.73 mW cm^–2^ and robust cycling stability. This work presents a simple method for using COFs as a smart platform to fabricate efficient electrocatalysts.

## 1. Introduction

The urgent need for renewable energy storage and conversion techniques has become increasingly apparent due to the serious energy crisis and environmental issues [1,2,3]. Over the last decade, the oxygen reduction reaction (ORR) has received considerable attention as a key process for sustainable and green energy storage and conversion technologies, such as fuel cells and rechargeable metal-air batteries [4,5,6,7]. However, the sluggish kinetics of oxygen at the cathode, due to significant activation energy barriers, often restricts its large-scale commercialization [8,9,10]. While platinum (Pt)-based nanomaterials are currently the most effective catalysts for ORR, their expensive price, poor durability, natural scarcity, and limited reserves hinder their widespread commercial use [11,12]. Therefore, there is an urgent and essential need to develop high-performance electrocatalysts.

Covalent organic frameworks (COFs) are a class of novel crystalline and porous polymer materials linked through strong covalent bonds [13,14,15,16,17,18,19,20]. Due to their highly ordered structures, tunable functionalities, and stability, COFs have garnered significant scientific interest for applications in various fields, including catalysis [21,22,23,24], energy storage [25,26], optoelectronics [27,28], and gas separation and capture [29], since their first reported use by the Yaghi group in 2005 [30]. Recently, COFs have also been widely explored and employed in electrochemical energy conversion systems, including ion conduction, lithium-ion batteries, and electrocatalysts, due to their well-defined electroactive sites and pre-designed active sites. Typically, a post-pyrolysis strategy is used to improve the catalytic performance of COFs towards ORR [31,32]. However, the high-temperature treatment process often leads to energy consumption and uncontrollable structural changes, resulting in poorly defined active sites that restrict a more detailed understanding of the ORR mechanism. As such, several pyrolysis-free COFs with precise skeleton structures and exact active sites have been explored as catalysts for ORR [33,34,35,36,37]. Despite these advances, the research on porphyrin-based COFs with various transition metals (Co, Ni, Fe) as electrocatalysts for ORR is still in its early stages due to the difficulty in synthesizing metal porphyrin monomers. Therefore, developing a general methodology to integrate various transition metals into COFs to improve catalytic ORR activity and explore the relationship between different metals and performance is highly anticipated and urgent.

Herein, a series of porphyrin-based COFs (TAPP-x-COF) with excellent ORR performance were constructed by precisely introducing various transition metals (Co, Ni, Fe) into periodic porphyrin-based COFs through Schiff-base condensation reaction and post-metallization. According to the experimental results, the catalytic ORR activity of TAPP-x-COF followed the trend of Co > Fe > Ni. Indeed, TAPP-Co-COF exhibited superior electrocatalytic performance with an onset potential of 0.80 V, a half-wave potential of 0.66 V vs. reversible hydrogen electrode (RHE), and a low Tafel slope of 73 mV dec^−1^, which outperformed other metal or pyrolysis-free COF-based electrocatalysts reported thus far.

## 2. Results and Discussions

**Figure 1:**
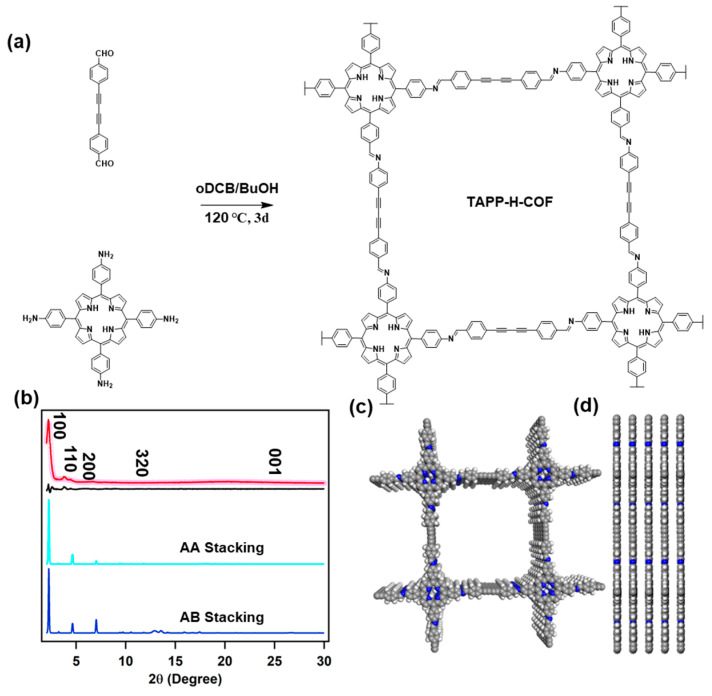
(**a**) Schematic of the synthesis of TAPP-H-COF. (**b**) Observed PXRD pattern (red) and profiles simulated using the Pawley refinement (pink circles) and their difference (black), AA-stacking (cyan), and staggered AB-stacking (blue) modes of the TAPP-H-COF. Model structures of TAPP-H-COF: (**c**) Top view and (**d**) Side view.

As shown in Figure 1a, TAPP-H-COF was first synthesized using 4,4′-(1,3-Butadiyne-1,4-diyl)bis[benzaldehyde] (BDB) as linker and 5,10,15,20-Tetrakis(4-aminophenyl)-21H,23H-porphine (TAPP) as knot catalyzed by 6M acetic acid under solvothermal condition. TAPP-H-COF was first characterized by Fourier-transform infrared (FT-IR) spectroscopy (Supplementary Material, Figure S1), which revealed a peak at 1699 cm^−1^ from the CHO of the monomer and a new absorption peak around 1622 cm^−1^ for the C=N vibration bond, confirming the successful formation of imine linkages in TAPP-H-COF. Additionally, characteristic C≡C and H-C≡C vibration bands were observed at 2120 and 3290 cm^−1^, respectively, suggesting the presence of ethynyl units in TAPP-H-COF. Thermogravimetric analysis (TGA) revealed no significant weight loss before 400 °C for TAPP-H-COF in N_2_ atmosphere (Supplementary Materials, Figure S2).

The crystalline structures of TAPP-H-COF were investigated using powder X-ray diffraction (PXRD) experiment. The diffraction curve of TAPP-H-COF exhibited four peaks at 2.24, 5.83, 12.59, and 25.05°, which were assigned to the 100, 110, 200, 320, and 001 facets, respectively (Figure 1b). Density-functional tight-binding (DFTB) calculations were applied to investigate the corresponding detailed crystal model. Obviously, the diffraction patterns calculated from the AA eclipsed stacking model matched well with the experimental PXRD curves, indicating the AA layer stacking structure in TAPP-H-COF (Figure 1b, pink circles). The PXRD curve from Pawley refinements against the experimental PXRD pattern exhibited good agreement and negligible residue, (*R*_wp_, *R*_p_) values of (4.91%, 4.38%) for TAPP-H-COF, where cell parameters were built by Pawley refinements using triclinic space group: a = 35.02 Å, b = 34.97 Å, c = 3.43 Å, *α* = *β* = *γ* = 90°. In addition, the PXRD pattern obtained from Pawley refinement is in good agreement with the experimental data of TAPP-H-COF supported by the negligible difference (Figure 1b, black curve). In contrast, the PXRD curve calculated from AB eclipsed stacking model showed obvious difference compared with the experimental data of TAPP-H-COF (Figure 1b, blue curve). Additionally, the unit cell structures from top and side view were built and are shown in Figure 1c,d.

**Figure 2:**
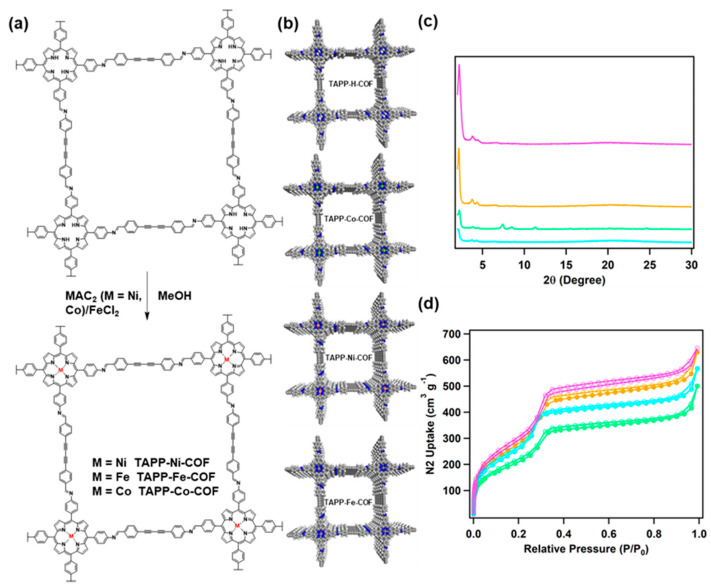
(**a**) Schematic of the synthesis of TAPP-x-COF. (**b**) Unit cell structures of TAPP-H-COF and TAPP-x-COF in top view. (**c**) Powder XRD patterns of TAPP-H-COF (pink), TAPP-Co-COF (green), TAPP-Fe-COF (cyan), and TAPP-Ni-COF (orange). (**d**) Nitrogen adsorption (filled circles)–desorption (open circles) isotherms.

Subsequently, the post-modification of TAPP-x-COF (Co, Ni, Fe) was carried out by directly metalating TAPP-H-COF with transition metal acetates (Co, Ni) or chlorate (Fe) via a heat-assisted method using ethanol as the solvent (50 °C) overnight (Figure 2a). After the incorporation of different metals (Co, Ni, Fe) into the TAPP-H-COF frameworks (Figure 2b), no obvious change was found in PXRD curves, and the only observable change was the slightly decreased peak intensity for TAPP-x-COF, confirming the retention of COFs structures (Figure 2c). The accessible porosities of the as-obtained TAPP-H-COF and TAPP-x-COF were studied by the N_2_ sorption measurement at 77 K. The Brunauer–Emmett–Teller (SBET) surface areas were calculated to be 1154 m^2^ g^−1^ for TAPP-H-COF. After post-metallization, a slightly decreased SBET was found for TAPP-x-COF (Co, Ni, Fe), whereas the SBET was 808 m^2^ g^−1^ for TAPP-Co-COF, 1129 m^2^ g^−1^ for TAPP-Ni-COF, and 1098 m^2^ g^−1^ for TAPP-Fe-COF, respectively (Figure 2d). The corresponding Langmuir BET was 2473, 2342, 2083, and 1762 m^2^ g^−1^ for TAPP-H-COF, TAPP-Ni-COF, TAPP-Fe-COF, and TAPP-Co-COF, respectively. Additionally, the pore size distribution calculated using non-local density functional theory (NLDFT) with Cylinder vs. N_2_-cylindrical pores-oxide surface model shows that TAPP-H-COF and TAPP-x-COF exhibit same homogeneous pore with a pore size of 3.58 nm (Supplementary Materials, Figure S3).

**Figure 3:**
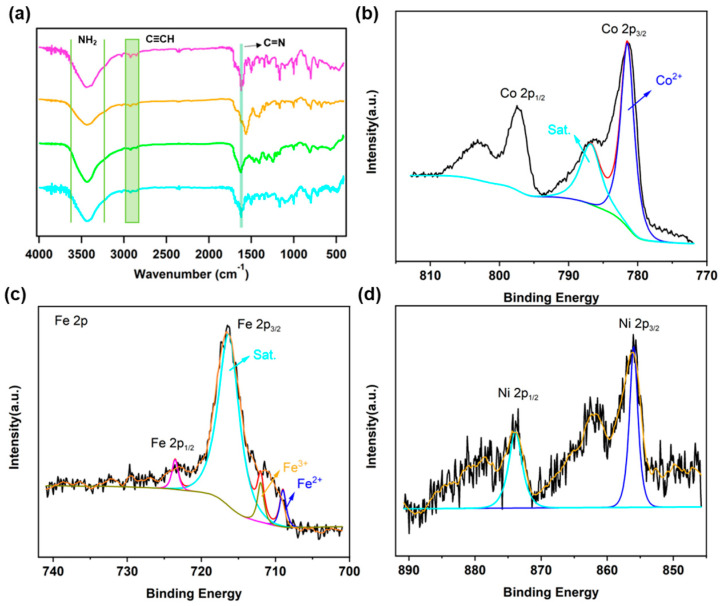
(**a**) FT-IR spectra of TAPP-H-COF (pink), TAPP-Co-COF (green), TAPP-Fe-COF (cyan), and TAPP-Ni-COF (orange). High-resolution XPS spectra of Co 2p for TAPP-Co-COF (**b**), Fe 2p for TAPP-Fe-COF (**c**), and Ni 2p for TAPP-Ni-COF (**d**).

After post-metallization, FT-IR spectra of TAPP-x-COF (Co, Ni, Fe) were found to be in good agreement with TAPP-H-COF, with the C=N vibration bond retained (Figure 3a). The TGA curves of TAPP-x-COF revealed that these metal-coordinated COFs remained stable up to 400 °C, with no significant differences observed before or after metal coordination (Supplementary Material, Figure S2). Furthermore, field-emission scanning electron microscopy (FE-SEM) was used to investigate the morphology of TAPP-H-COF and TAPP-x-COF, where flower-like nanoclusters were found for TAPP-H-COF and TAPP-x-COF. In addition, after post-metallization, no obvious change was found in the morphology, further indicating that the ordered structures were retained. Moreover, EDX analysis was applied to investigate the elemental distribution for TAPP-H-COF and TAPP-x-COF, where the elements of these COFs were distributed uniformly over the COFs (Supplementary Material, Figures S5–S8).

The total Fe, Ni, and Co content in TAPP-x-COF was detected to be 4.3 wt%, 4.6 wt% and 4.2 wt%, respectively, by inductively coupled plasma optical emission spectrometer (ICP-OES) analyses. The coordination of metal ions into TAPP-H-COF was further demonstrated by the X-ray photoelectron spectroscopy (XPS) measurements (Figure 3 and Figure S9). Taking TAPP-Co-COF as an example, the observed Co 2p3/2 binding energy of 781.1 eV and 2p1/2 binding energy of 796.2 eV were assigned to Co (II) (Figure 3b), indicating the successful modification of Co ions into the structure. Meanwhile, compared with N 1s XPS spectrum of TAPP-H-COF, an obvious upshift in N 1s peaks in TAPP-Co-COF was detected (Supplementary Materials, Figure S9), which could also be attributed to the coordination of nitrogen atoms to Co ions.

**Figure 4:**
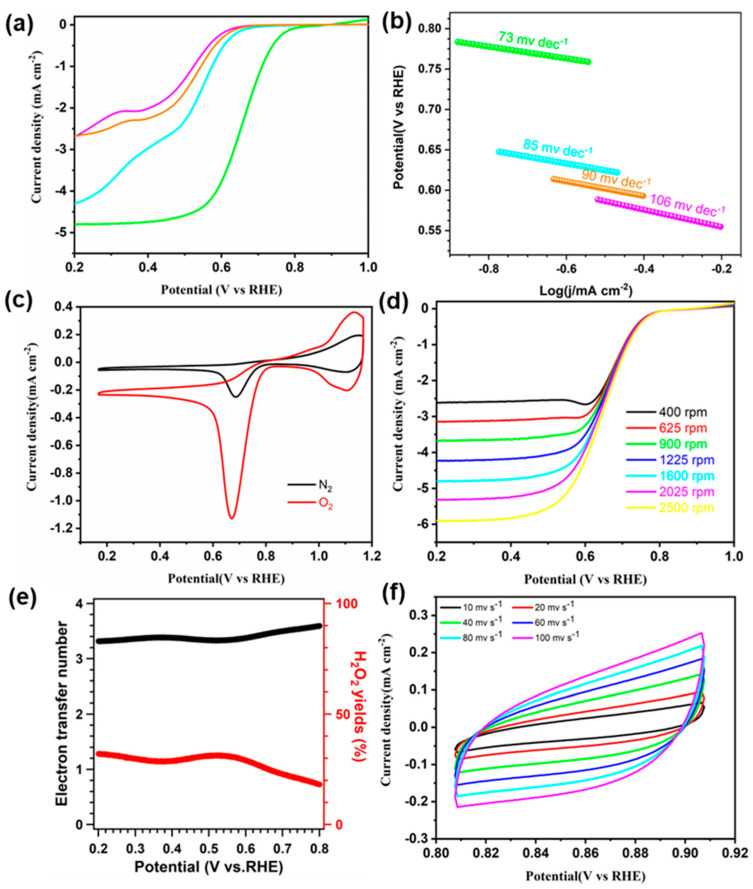
(**a**) ORR polarization curves and (**b**) Tafel plots of TAPP-H-COF (pink), TAPP-Co-COF (green), TAPP-Fe-COF (cyan), and TAPP-Ni-COF (orange). (**c**) CV curves of TAPP-Co-COF in N_2_- and O_2_-saturated 0.1M KOH. (**d**) ORR polarization curves of TAPP-Co-COF at different rotating speeds. (**e**) Electron transfer number and H_2_O_2_ yield plots calculated from the RRDE measurements over TAPP-Co-COF in O_2_-saturated KOH (0.1 M) aqueous solution. (**f**) Cyclic voltametric curves of TAPP-Co-COF in 0.1 M KOH solution at different scan rates (10, 20, 40, 60, 80, and 100 mV s^−1^).

The electrocatalytic ORR performance levels of the obtained TAPP-x-COF were evaluated by a three-electrode system in an O_2_-saturated 0.1 M KOH electrolyte with a sweep of 1600 rpm at room temperature. As depicted in Figure 4a, the linear sweep voltammetry (LSV) curves of the samples displayed that TAPP-Co-COF exhibited the best ORR performance when compared to that of TAPP-Ni-COF, TAPP-Fe-COF, and TAPP-H-COF, which clearly indicates the importance of introducing Co atoms into the skeleton for improving ORR activity. The half-wave potential of TAPP-Co-COF is 0.66 V vs. RHE, which is more positive than those of TAPP-Ni-COF (0.52 V vs. RHE), TAPP-Fe-COF (0.51 V vs. RHE), and TAPP-H-COF (0.50 V vs. RHE). Moreover, the TAPP-Co-COF has the highest onset potential of up to 0.80 V vs. RHE and current density of around 4.82 mA cm^−2^ among these COFs. As shown in Figure 4b, the Tafel slope of TAPP-Co-COF was determined to be 73 mV dec^−1^, which is smaller than those of TAPP-Fe-COF (85 mV dec^−1^), TAPP-Ni-COF (90 mV dec^−1^), and TAPP-H-COF (106 mV dec^−1^), thus revealing the faster ORR kinetics for TAPP-Co-COF. Obviously, the changing trend in obtained Tafel slope values is consistent with the result of LSV curves. The electrocatalytic ORR activity of COFs was verified from the oxygen reduction peak at 0.68 V vs. RHE for TAPP-Co-COF, 0.54 V vs. RHE for TAPP-Fe-COF, 0.52 V vs. RHE for TAPP-Ni-COF, and 0.51 V vs. RHE for TAPP-H-COF by cyclic voltammetry (CV) (Figure 4c and Figure S10). In addition, the LSV curves were obtained at scan rates from 400 to 1600 rpm (Supplementary Materials, Figures S4d and S11).

Additionally, the electrochemical impedance spectroscopy (EIS) measurements were performed on the obtained COFs to evaluate their conductive properties. Accordingly, the charge transfer resistance for TAPP-Co-COF, TAPP-Fe-COF, TAPP-Ni-COF, and TAPP-H-COF was obtained to be 135, 190, 267, and 331 Ω, respectively (Figure S12), which indicates that TAPP-Co-COF possesses a much faster electron transfer capability. The electron transfer numbers (n) for TAPP-Co-COF were higher than 3.2 from 0.2 to 0.8 V (Figure 4e). Additionally, the corresponding H_2_O_2_ yields less than 29.39%, suggesting a 4e- pathway in the ORR process.

A linear relationship between electrochemically active surface areas (ECSAs) and double-layer capacitance (Cdl) is well known. Consequently, Cdl was performed via the CV method (Figure 4e and Figure S13). As shown in Figure 4f, the Cdl value for TAPP-Co-COF was obtained, being 1.36 mF cm^−2^, and the value is larger than that of 0.22 mF cm^−2^ for TAPP-Fe-COF and 0.10 mF cm^−2^ for TAPP-Ni-COF, as well as 0.08 mF cm^−2^ for TAPP-H-COF (Figure S14), indicating that the TAPP-Co-COF can afford more efficient active sites. The long-term stability for TAPP-H-COF, TAPP-Ni-COF, TAPP-Fe-COF, and TAPP-Co-COF towards ORR showed the current density was 87.1%, 84.8%, 87.3%, and 93.7%, respectively, after 20000 s (Supplementary Materials, Figure S15), indicating its excellent long-term stability, especially for TAPP-Co-COF.

**Figure 5:**
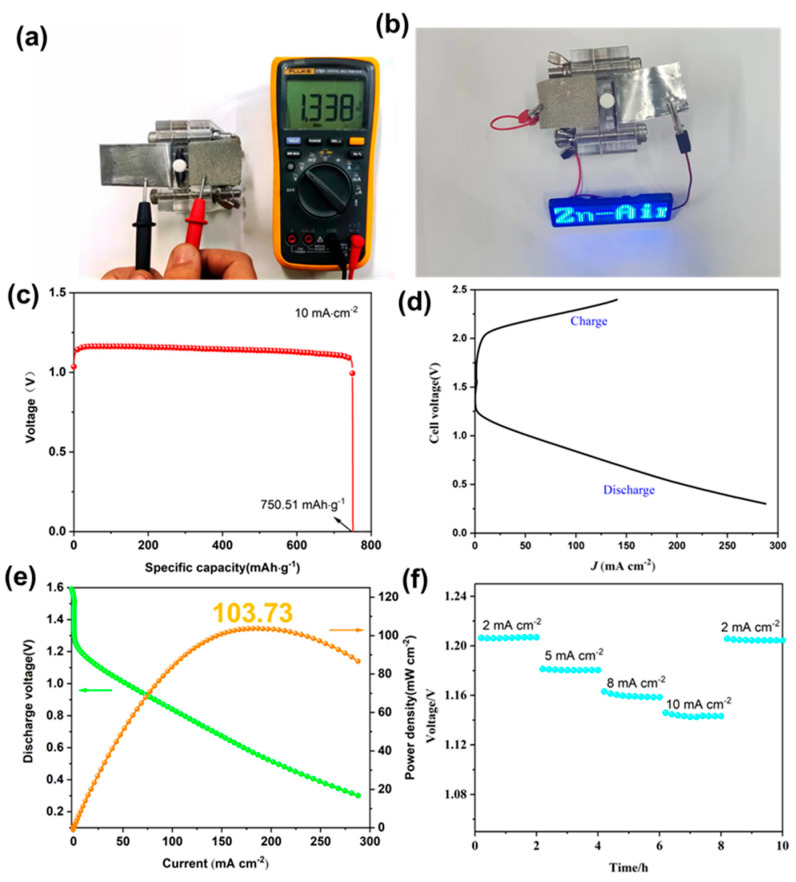
(**a**) OCV measurement of Zn–air battery. (**b**) Photographs of LEDs powered by Zn–air batteries with TAPP-Co-COF as the air-cathode catalyst. (**c**) Specific capacities for TAPP-Co-COF at a current density of 10 mA cm^−2^. (**d**) Charge–discharge profiles of TAPP-Co-COF based ZAB. (**e**) The discharge polarization plots and power density diagrams of Zn–air batteries. (**f**) The discharge curves of assembled Zn–air batteries at current densities of 2.0, 5.0, 8.0, and 10 cm^−2^.

To evaluate the practical application of TAPP-Co-COF as an air-cathode catalyst, a Zn–air battery was assembled. The battery consisted of a Zn-foil anode and an air cathode loaded with catalysts on carbon paper. The TAPP-Co-COF electrode exhibited an open circuit voltage (OCV) of 1.338 V (Figure 5a), and the Zn–air batteries assembled using TAPP-Co-COF successfully powered light-emitting diodes (LEDs) (Figure 5b). The specific capacity of the TAPP-Co-COF electrode was calculated to be 750.51 mA h g^−1^_Zn_ (Figure 5c). Long-term discharge curves at 10 mA cm^−2^, until the battery stopped functioning with Zn foil being completely consumed, were also obtained (Figure 5d). The power density of TAPP-Co-COF reached a maximum of 103.73 mW cm^−2^ when the current density was 182 mA cm^−2^ (Figure 5e). Additionally, the discharge curves of the assembled Zn–air battery at current densities of 2.0, 5.0, 8.0, and 10 mA cm^−2^ were investigated, and TAPP-Co-COF consistently maintained a higher discharge voltage (Figure 5f), indicating its promising application in Zn–air batteries. These results demonstrate the potential of TAPP-Co-COF as an efficient air-cathode catalyst for practical applications in Zn–air batteries.

## 3. Materials and Methods

*Electrocatalytic measurements.* The ORR catalyst (4 mg; COFs) and active carbon (1 mg) were dispersed in a Nafion ethanol solution (0.25 wt%, 500 μL) and were sonicated for 2 h to yield a homogeneous ink. The catalyst ink (12 mL) was pipetted onto a glassy carbon electrode (d = 5.00 mm, S = 0.196 cm^2^) with a loading of 0.6 mg cm^−^^2^. The commercially available 20 wt% platinum on carbon black (Pt/C, BASF) was measured for comparison. The Pt/C sample (5 mg) was dispersed in a Nafion solution (0.25 wt%, 500 mL) by sonication for 2 h to obtain a well-dispersed ink, and the catalyst ink (9 mL) was pipetted onto the glassy carbon electrode surface.

*ORR performance tests.* All the electrochemical measurements were conducted in a conventional three-electrode cell using the PINE electrochemical workstation (Pine Research Instrumentation, United States (USA)) at room temperature. The Ag/AgCl (3m KCl) and platinum wire were used as reference and counter electrodes, respectively. A rotating ring disk electrode (RRDE) with a Pt ring and a glassy carbon disk served as the substrate for the working electrode for evaluating the ORR activity and selectivity of various catalysts. The electrochemical experiments were conducted in O_2_-saturated aqueous solution of KOH (0.1 M) for the ORR. The Tafel slope was estimated by linear fitting of the polarization curves according to the Tafel equation (h = b × logj + a, in which j is the current density and b is the Tafel slope). For the cyclic voltammetry (CV) tests, the potential range was circularly scanned between −0.8 and 0 V at a scan rate of 50 mV s^−^^1^ after purging O_2_ gas for 30 min. For estimation of the double-layer capacitance, the electrolyte was deaerated by bubbling with nitrogen, and then the voltammogram was evaluated again in the deaerated electrolyte. The rotating disk electrode (RDE) measurements were conducted at different rotation rates from 400 to 1600 rpm at a scan rate 10 mV s^−^^1^.

## 4. Conclusions

In summary, a series of porphyrin-based COFs with various metals were designed and synthesized by reversible imine condensation and post-metallization. These COFs exhibited high crystallinity, excellent porosity, and efficient ORR performance. In particular, TAPP-Co-COF exhibited a superior activity for ORR with an onset potential of 0.80 V (vs. RHE), a half-wave potential of 0.66 V (vs. RHE), and low Tafel slope of 73 mV dec^−1^. The ORR performance of TAPP-Co-COF is comparable with the previously reported COF materials without pyrolysis. Theoretical and experimental results demonstrated that the Co atoms in the skeleton of COFs could serve as highly active sites for electrocatalytic ORR.

## Data Availability

No data was shared.

## Supplementary Materials

The following supporting information can be downloaded at: https://www.mdpi.com/article/10.3390/molecules28124680/s1. Figures S1–S15; Tables S1 and S2.
