# Supplementary material for: Modulating the Oxygen Reduction Reaction Performance via Precisely Tuned Reactive Sites in Porphyrin-Based Covalent Organic Frameworks
Source: Molecules. 2023 Jun 9;28(12):4680. doi: 10.3390/molecules28124680 (PMC10301357; doi:10.3390/molecules28124680)
Supplement: Supplementary file 1 [file molecules-28-04680-s001.zip › molecules-2418115-supplementary.pdf]

Modulating the Oxygen Reduction Reaction Performance via Precisely Tuned Reactive Sites in Porphyrin-Based Covalent Organic Frameworks

Contents

Section A. Materials and Methods

Section B. Synthetic Procedures

Section C. Supporting Figures

Section D. Supporting Tables

Section A. Materials and Methods

Characterization. ^1H NMR spectra were measured on a Bruker 400 MHz spectrometer, while chemical shifts (δ in ppm) were determined using a standard of the solvent residual proton. ICP-MS was carried out on a Perkin-Elmer Elan DRC II quadrupole inductively coupled plasma mass spectrometer analyzer. Fourier transform infrared (FT IR) spectra were recorded on a JASCO model FT IR-6100 infrared spectrometer. X-ray diffraction (XRD) data were recorded on a Bruker D8 Focus Powder X-ray Diffractometer by using powder on glass substrate, from 2θ = 1.5° up to 30° with 0.01°increment. Elemental analysis was performed on an Elementar vario MICRO cube elemental analyzer. TGA measurements were performed on a Discovery TGA under N_2, by heating from 30 to 800 °C at a rate of 10 °C min^{-1}. Nitrogen sorption isotherms were measured at 77 K with a TriStar II instrument (Micromeritics). The Brunauer–Emmett–Teller (BET) method was utilized to calculate the specific surface areas. By using the non-local density functional theory (NLDFT) model, the pore volume was derived from the sorption curve. X-ray photoelectron spectroscopy (XPS) experiments were performed on a Thermo Scientific K-Alpha XPS spectrometer using an AlKa X-ray radiation source. Morphology images were characterized with a Zeiss Merlin Compact filed emission scanning electron microscope (FE-SEM) equipped with an energy-dispersive X-ray spectroscopy (EDS) system at an electric voltage of 5 KV. The ICP-OES measurements were performed on a Thermo Scientific iCAP 7400 instruments.

Computational calculations. The crystalline structure of COFs was determined using the density-functional tight-binding ($DFTB^+$) method including Lennard-Jones (LJ) dispersion. The calculations were carried out with the $DFTB^+$ program package version 1.2. DFTB is an approximate density functional theory method based on the tight binding approach and utilizes an optimized minimal LCAO Slater-type all-valence basis set in combination with a two-center approximation for Hamiltonian matrix elements. The Coulombic interaction between partial atomic charges was determined using the self-consistent charge (SCC) formalism. Lennard-Jones type dispersion was employed in all calculations to describe van der Waals (vdW) and π-stacking interactions. The lattice dimensions were optimized simultaneously with the geometry. Standard DFTB parameters for X–Y element pair (X, Y = C, H, N, and S) interactions were employed from the mio-0-1 set.

Electrocatalytic measurements. The ORR catalyst (4 mg; COFs) and active carbon (1 mg) were

dispersed in a Nafion ethanol solution (0.25 wt %, 500 μL) and were sonicated for 2 h to yield a homogeneous ink. The catalyst ink (12 mL) was pipetted onto a glassy carbon electrode (d=5.00 mm, S=0.196 cm^2) with a loading of 0.6 mg cm^{-2}. The commercially available 20 wt % platinum on carbon black (Pt/C, BASF) was measured for comparison. The Pt/C sample (5 mg) was dispersed in a Nafion solution (0.25 wt %, 500 mL) by sonication for 2 h to obtain a well-dispersed ink, and the catalyst ink (9 mL) was pipetted onto the glassy carbon electrode surface.

ORR performance tests. All the electrochemical measurements were conducted in a conventional three-electrode cell using the PINE electrochemical workstation (Pine Research Instrumentation, USA) at room temperature. The Ag/AgCl (3m KCl) and platinum wire were used as reference and counter electrodes, respectively. A rotating ring disk electrode (RRDE) electrode with a Pt ring and a glassy carbon disk served as the substrate for the working electrode for evaluating the ORR activity and selectivity of various catalysts. The electrochemical experiments were conducted in O_2-saturated aqueous solution of KOH (0.1 M) for the ORR. The Tafel slope was estimated by linear fitting of the polarization curves according to the Tafel equation (h=b*logj+a, in which j is the current density and b is the Tafel slope). For the cyclic voltammetry (CV) tests, the potential range was circularly scanned between -0.8 and 0 V at a scan rate of 50 mV s^{-1} after purging O_2 gas for 30 min. For estimation of the double layer capacitance, the electrolyte was deaerated by bubbling with nitrogen, and then the voltammogram was evaluated again in the deaerated electrolyte. The rotating disk electrode (RDE) measurements were conducted at different rotation rates from 400 to 1600 rpm at a scan rate 10 mV s^{-1}.

Zn-air battery test. A home-made ZAB was employed for the evaluation of the battery performance. A polished zinc foil was used as the anode, and a hydrophilic carbon fiber paper substrate coating with the catalyst layer (1 mg·cm^{-2}) was used as the air cathode. The mixed solution of 6 M KOH + 0.2 M $Zn(CH_3COO)_2 \cdot 2H_2O$ was used as the electrolyte in the alkaline zinc-air batteries. The LSV polarization curve measurements were performed at 10 mV·s^{-1} on a CHI660 electrochemical workstation at room temperature. The specific capacity was obtained by normalizing mAh to the mass of consumed Zn during the long-term discharge process by a LAND testing system for comparison, the alkaline Pt/C based ZABs were assembled by using the mixture of commercial Pt/C catalyst (loading of 1 mg·cm^{-2}) as the air electrode. Notably, all the

measurements of Zn-air batteries were conducted in air rather than pure oxygen stationary atmosphere.

Section B. Synthetic Procedures

5,10,15,20-tetrakis(4-aminophenyl)-21H,23H-porphine and 4,4'-(1,3-Butadiyne-1,4-diyl)bis[benzaldehyde] obtained from Jilin Chinese Academy of Sciences-Yanshen Technology Co., Ltd., All the other solvents were purchased from Aladdin Chemicals and used as received without further purification.

Synthesis of TAPP-H-COF. 5,10,15,20-tetrakis(4-aminophenyl)-21H,23H-porphine (TAPP 17.0 mg, 0.025 mmol) and 4,4'-(1,3-Butadiyne-1,4-diyl)bis[benzaldehyde] (13.0 mg, 0.05 mmol) were dissolved in a mixture solvent of 0.5 mL n-butanol and 1.5 mL 1,2-dichlorobenzene using a thick-walled pressure tube. Then, 200 μL of aqueous acetic acid (6 M) were added in the tube and the tube was ultrasonicated for 5 min. This mixture was heated up to 120 ℃ for a 3 day. The resulting precipitates were collected and washed with DMF and tetrahydrofuran for three times, and then dried under vacuum at 120 °C for 12 h. The target COF sample was achieved as red powder in a yield of approximately 82%.

Synthesis of TAPP-x-COF. In a typical procedure, as-prepared TAPP-H-COF (50mg) and the corresponding metal sources (200 mg) were dispersed in ethanol (50 mL) and stirred at 50 °C for overnight. The product was collected through centrifugation, washed with water, methanol, and acetone three times, and dried under vacuum for further use.

Section C. Supporting Figures

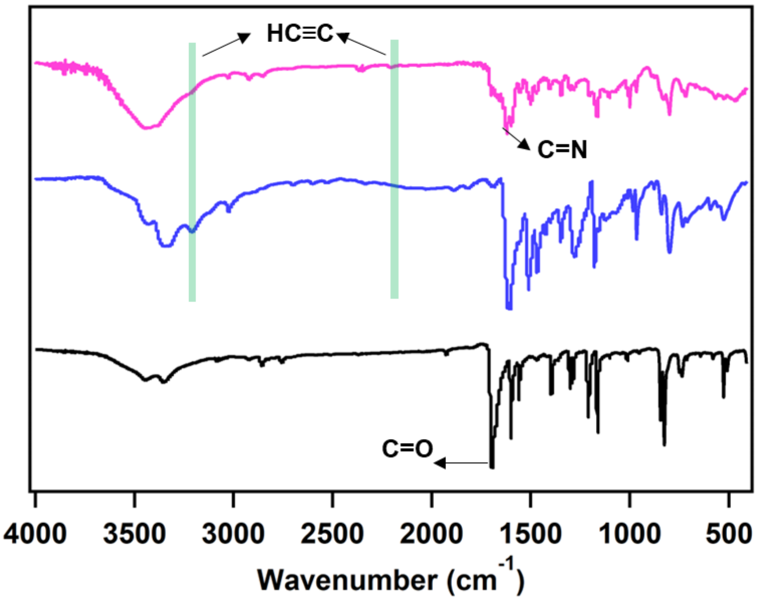

Figure S1. FT IR spectra of TAPP-H-COF (pink) and the corresponding monomers: BDB (blue) and TAPP (black).

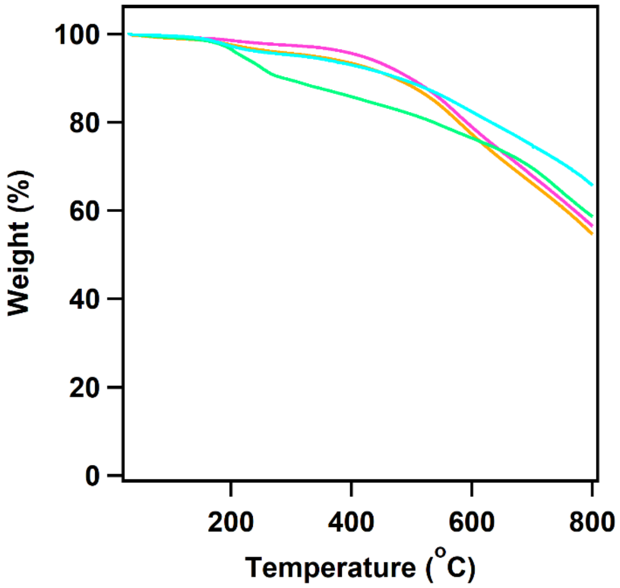

Figure S2. Thermogravimetric curves of TAPP-H-COF (red), TAPP-Co-COF (green), TAPP-Fe-COF (cyan), and TAPP-Ni-COF (orange).

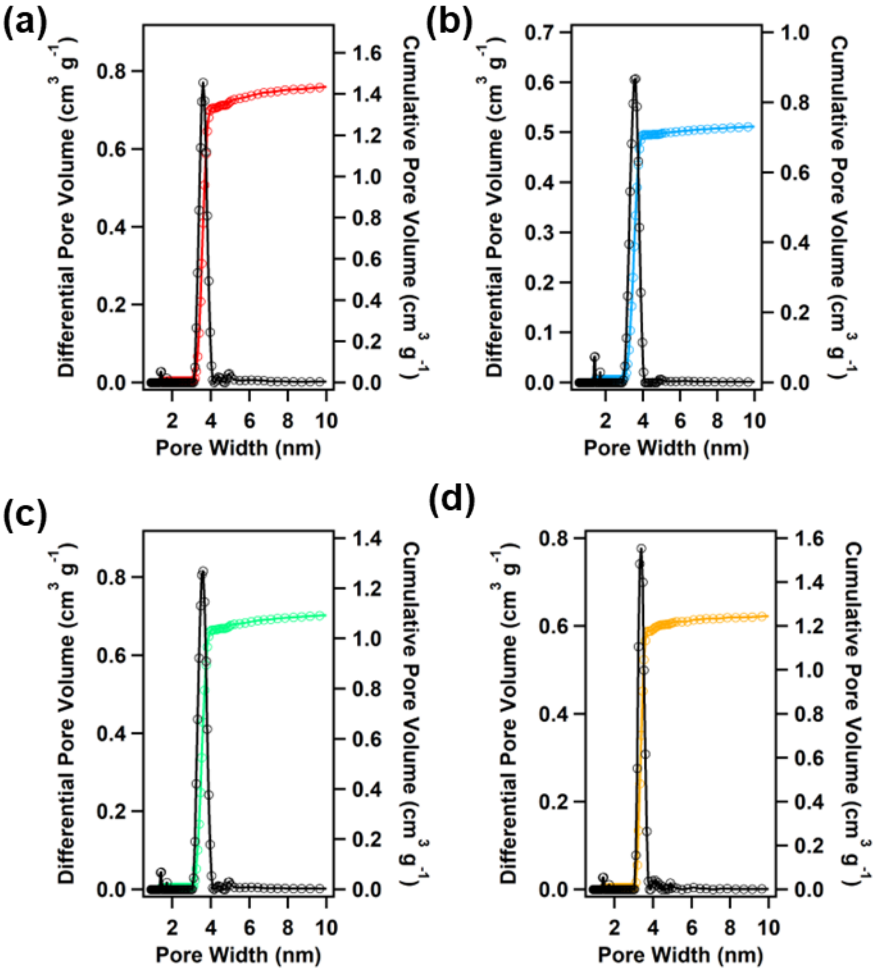

Figure S3. Pore size distribution of TAPP-H-COF (a), TAPP-Co-COF (b), TAPP-Fe-COF (c), and TAPP-Ni-COF (d).

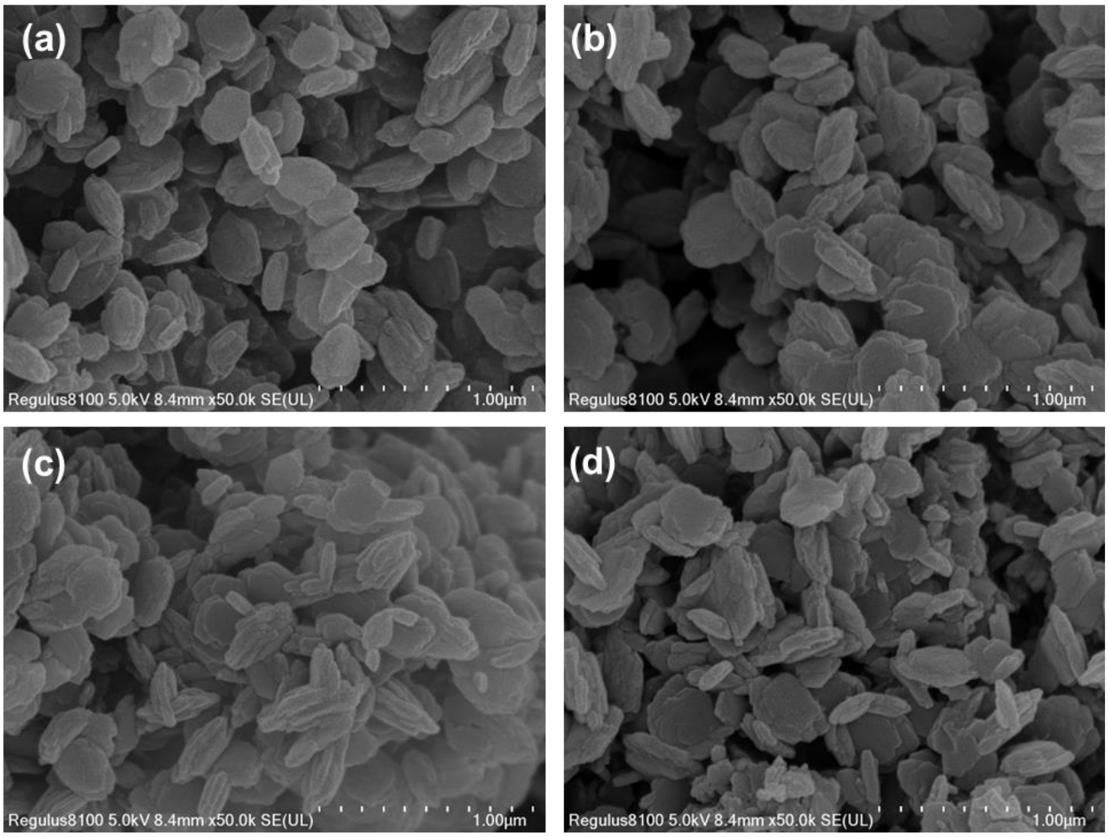

Figure S4. SEM images of TAPP-H-COF (a), TAPP-Co-COF (b), TAPP-Fe-COF (c), and TAPP-Ni-COF (d).

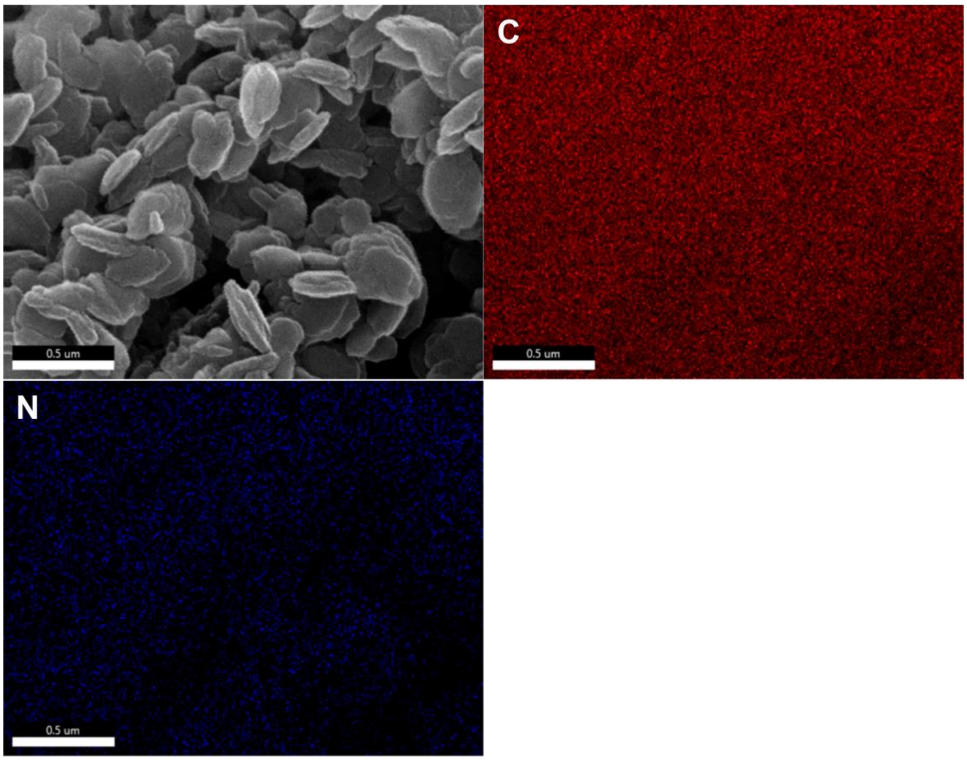

Figure S5. EDS mapping of TAPP-H-COF.

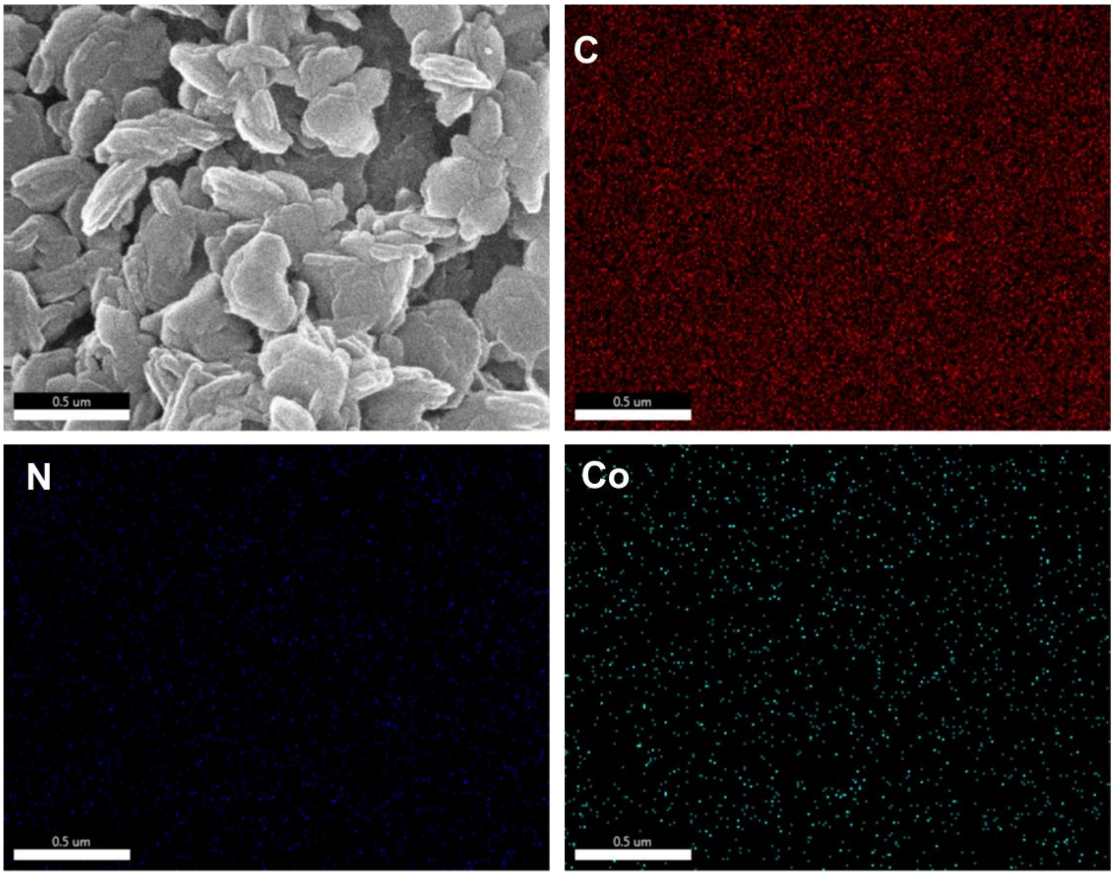

Figure S6. EDS mapping of TAPP-Co-COF.

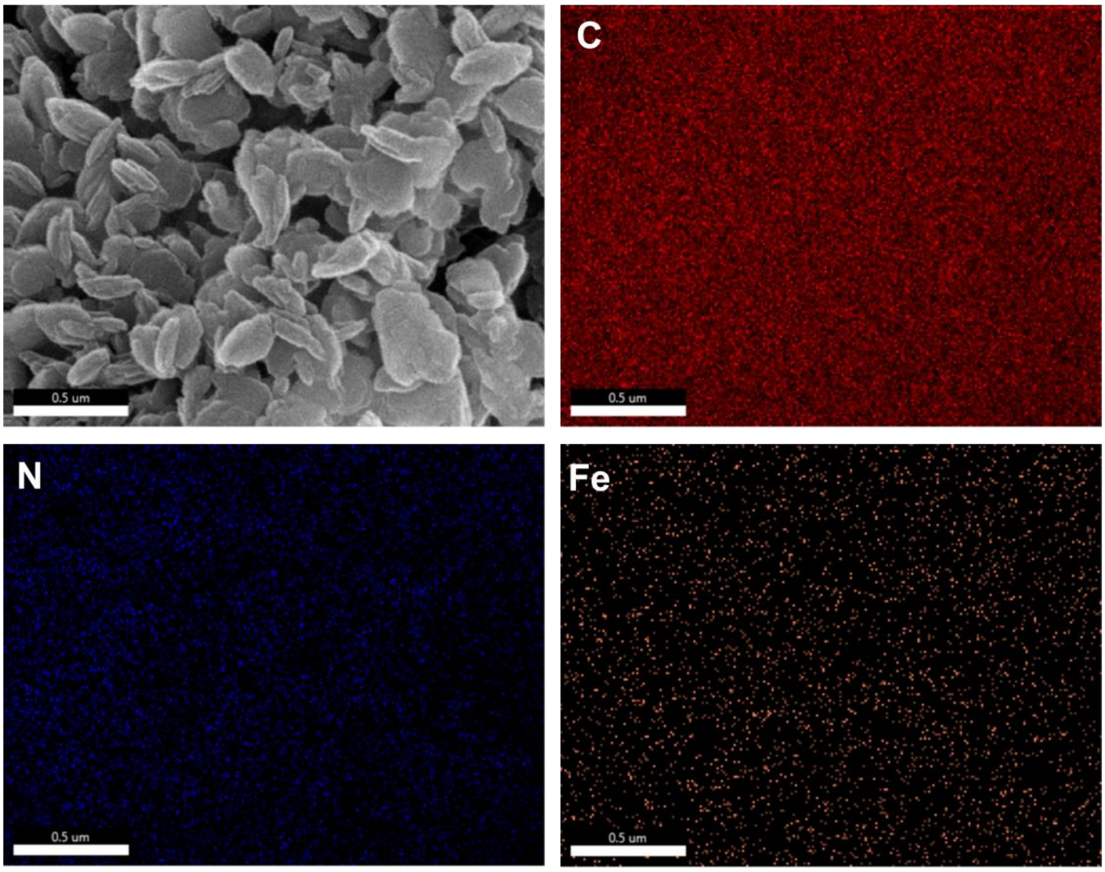

Figure S7. EDS mapping of TAPP-Fe-COF.

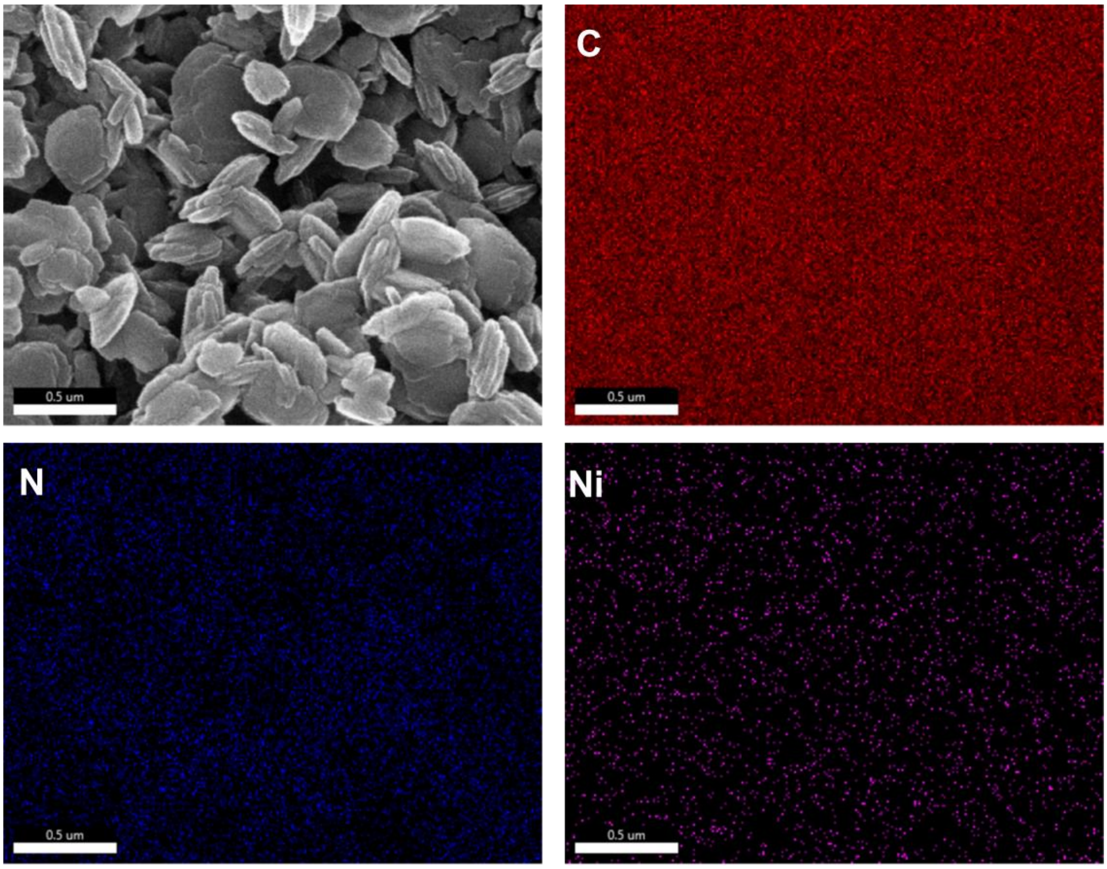

Figure S8. EDS mapping of TAPP-Ni-COF.

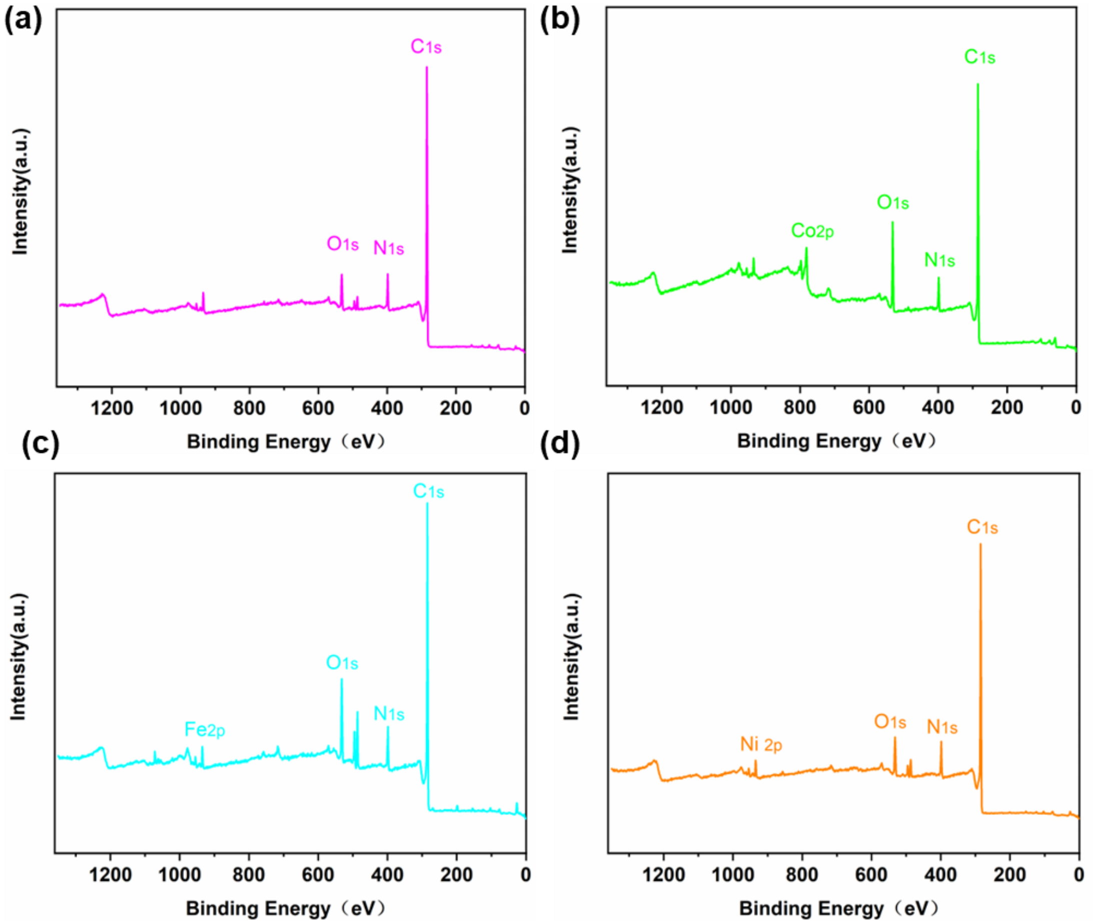

Figure S9. Survey XPS spectrum of TAPP-H-COF (a), TAPP-Co-COF (b), TAPP-Fe-COF (c), and TAPP-Ni-COF (d).

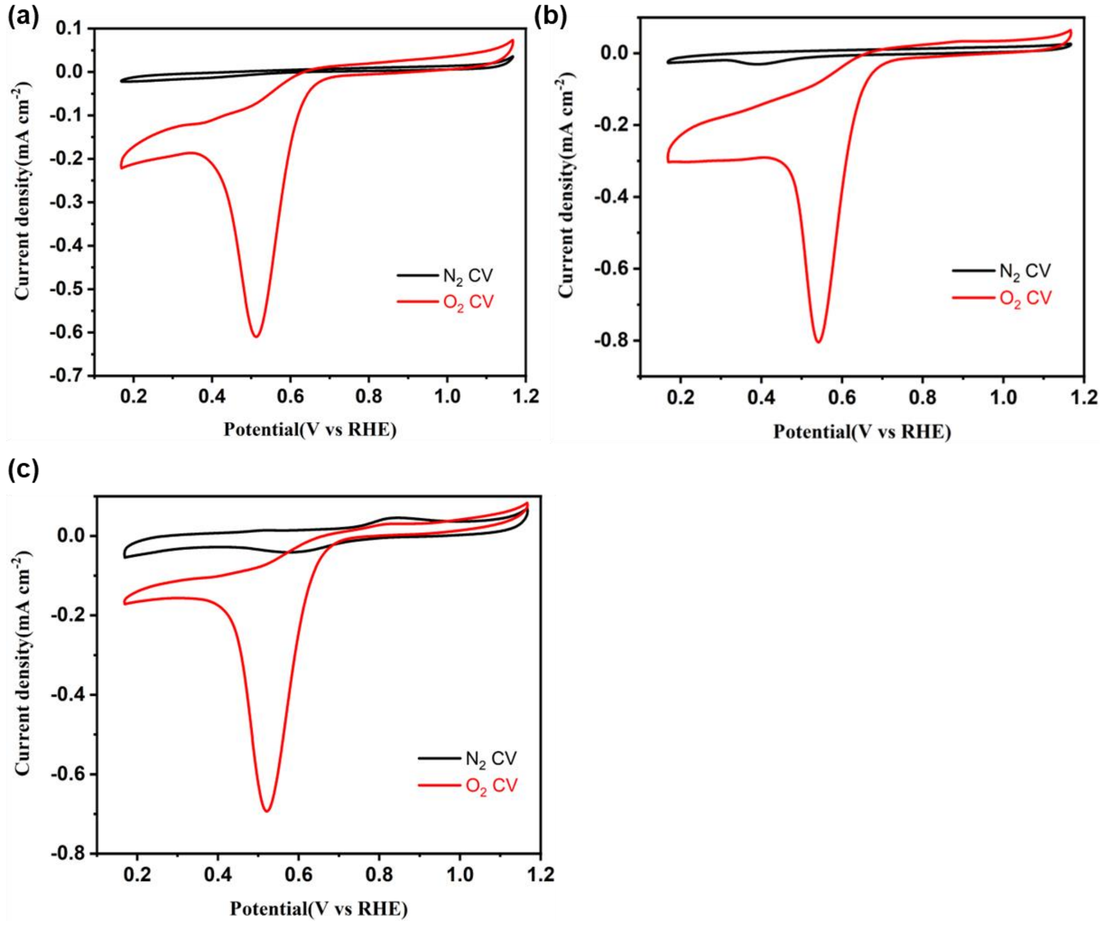

Figure S10. CV curves in N_2- and O_2-saturated 0.1m KOH of (a) TAPP-H-COF, (b) TAPP-Fe-COF, and (c) TAPP-Ni-COF.

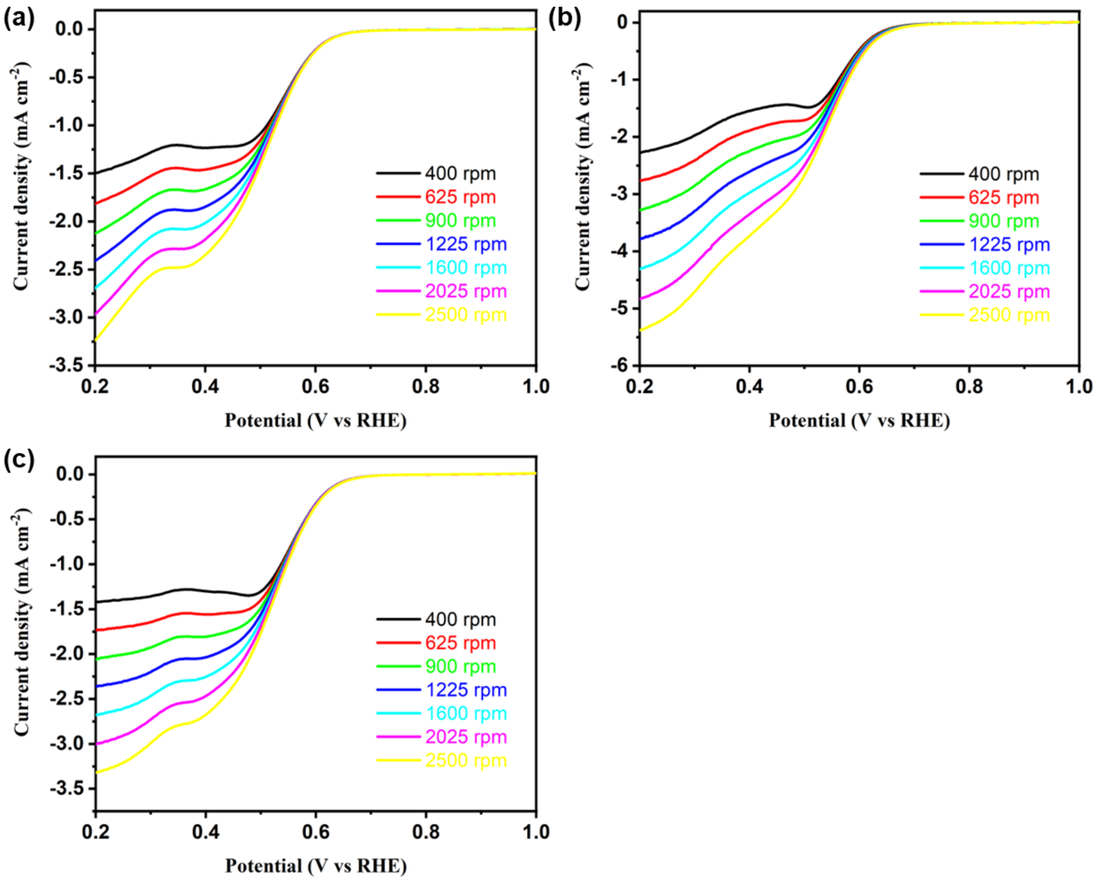

Figure S11. ORR polarization curves at different rotating speeds of (a) TAPP-H-COF, (b) TAPP-Fe-COF, and (c) TAPP-Ni-COF.

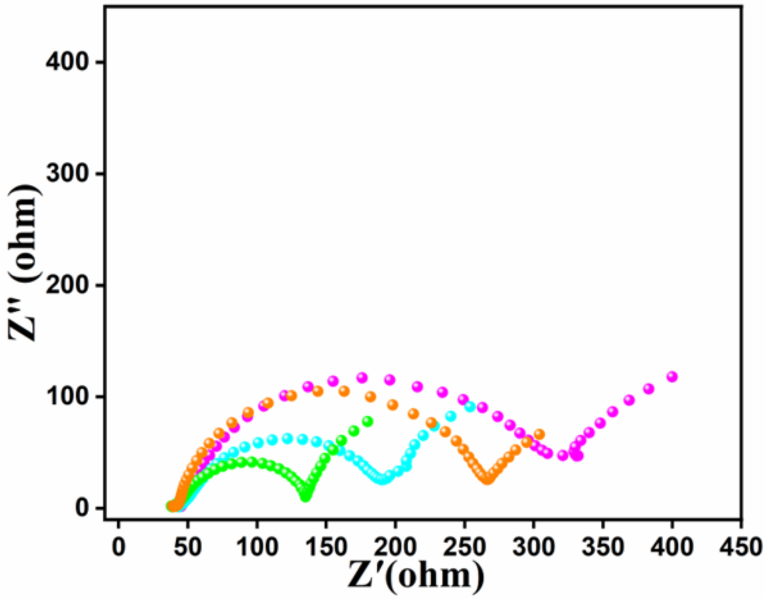

Figure S12. The impedance at open circuit voltage for TAPP-H-COF (pink), TAPP-Co-COF (green), TAPP-Fe-COF (cyan), and TAPP-Ni-COF (orange).

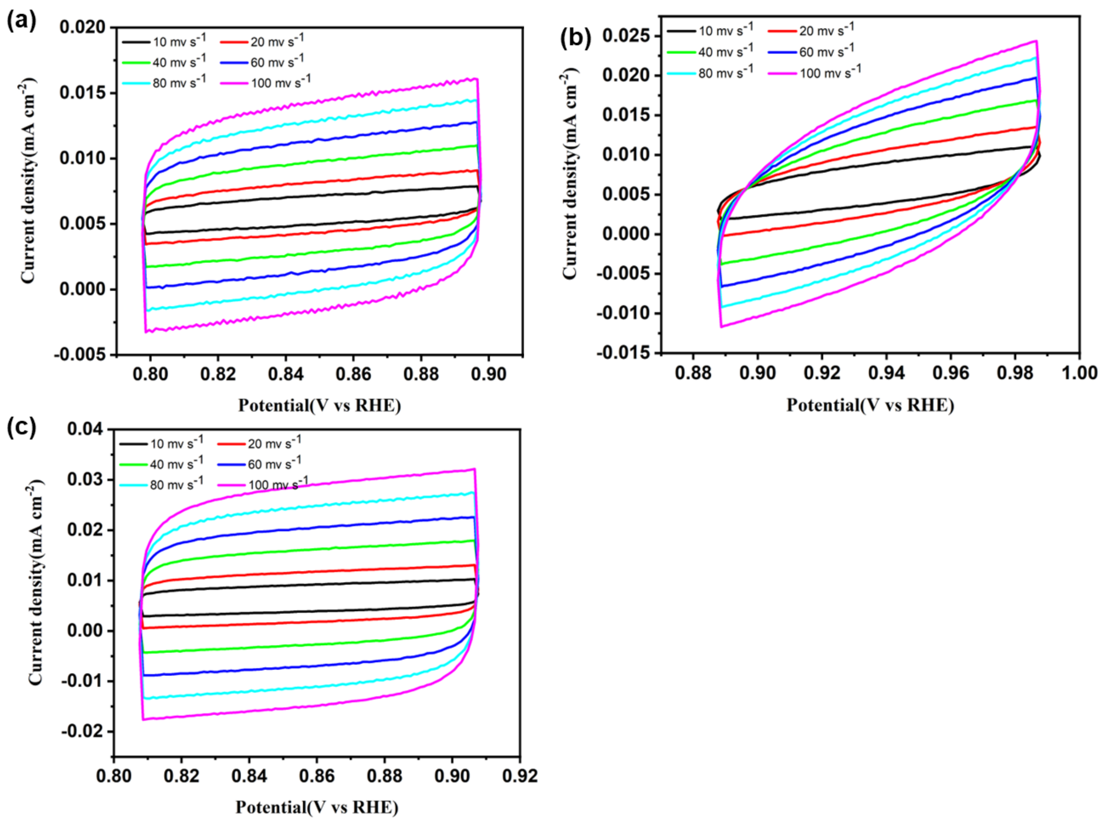

Figure S13. Cyclic voltametric curves of (a) TAPP-H-COF, (b) TAPP-Fe-COF, and (c) TAPP-Ni-COF in 0.1 M KOH solution at different scan rates (10, 20, 40, 60, 80 and 100 mV s^{-1}).

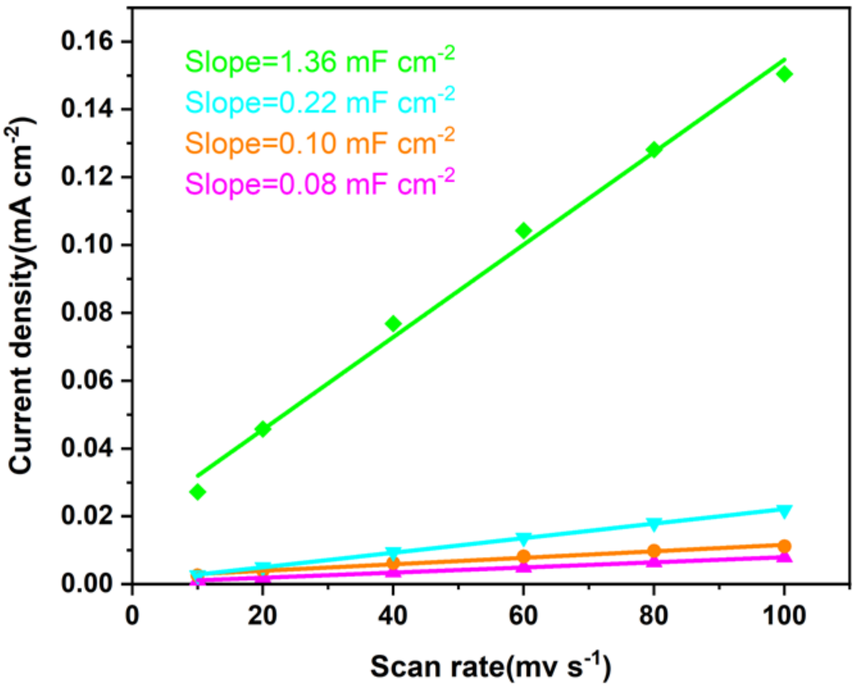

Figure S14. Cdl values for TAPP-H-COF (pink), TAPP-Co-COF (green), TAPP-Fe-COF (cyan), and TAPP-Ni-COF (orange).

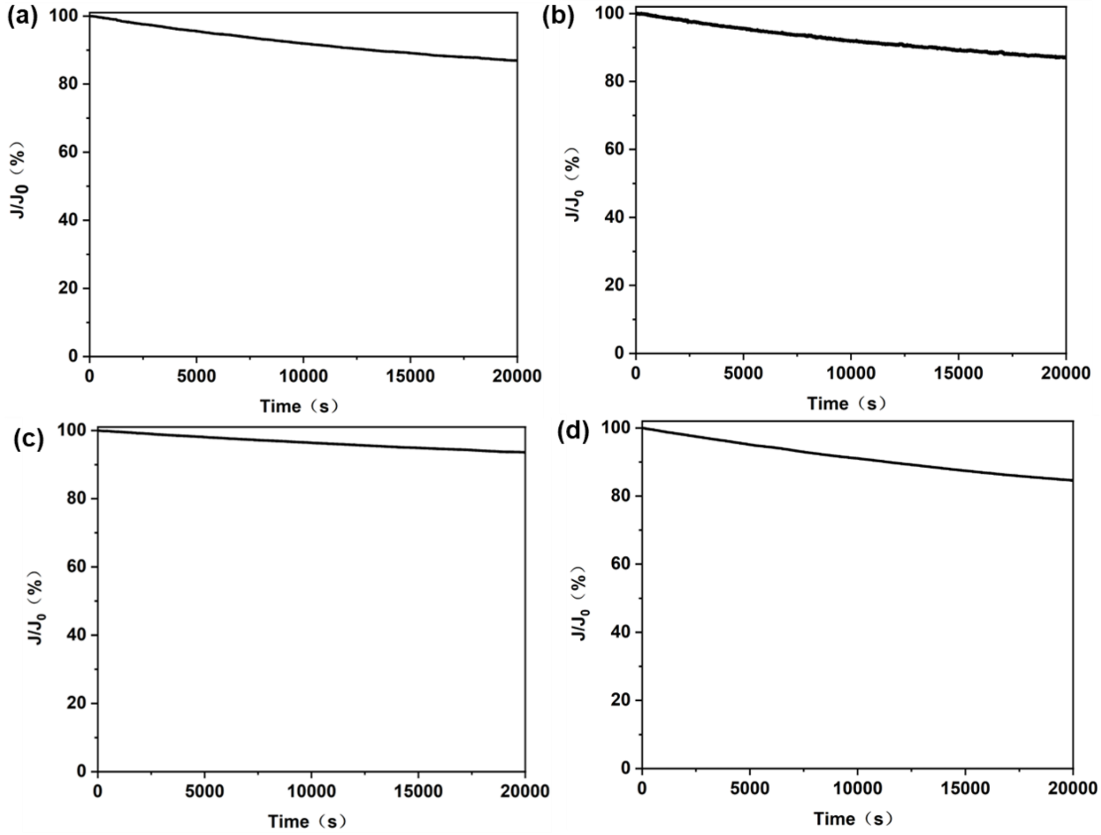

Figure S15. The durability stability of (a) TAPP-H-COF, (b) TAPP-Fe-COF, (c) TAPP-Co-COF, and (d) TAPP-Ni-COF in oxygen-saturated, KOH solution (0.1 M) aqueous solution for 20000s.

Section D. Supporting Tables

Table S1. Crystal data, data collection, and structure refinement parameters for TAPP-H-COF (Space group symmetry P1, a = 35.02 Å, b = 34.97 Å, c = 3.43 Å, $\alpha = \beta = \gamma = 90°$, R_p = 4.91% and R_{wp} = 4.38%.).

Atom	*x*	*y*	*z*
C1	0.43045	0.58951	0.5
C2	0.40488	0.56433	0.5
C3	0.42272	0.52905	0.5
N4	0.45948	0.53436	0.5
C5	0.46537	0.57104	0.5
C6	0.59332	0.56084	0.5
C7	0.56707	0.58749	0.5
C8	0.5338	0.57042	0.5
N9	0.53897	0.53366	0.5
C10	0.57565	0.5279	0.5
C11	0.49972	0.58993	0.5
C12	0.56717	0.39918	0.5
C13	0.59274	0.4243	0.5
C14	0.57488	0.45955	0.5
N15	0.53811	0.45431	0.5
C16	0.53222	0.41764	0.5
C17	0.59447	0.49352	0.5
C18	0.4043	0.42758	0.5
C19	0.43055	0.40087	0.5
C20	0.46379	0.41796	0.5
N21	0.45861	0.45473	0.5
C22	0.42195	0.46055	0.5
C23	0.40315	0.495	0.5
C24	0.49792	0.39861	0.5
C25	0.63698	0.4927	0.5
C26	0.50019	0.63244	0.5
C27	0.36065	0.49583	0.5
C28	0.4982	0.3561	0.5
C29	0.65594	0.46886	0.5
C30	0.69445	0.46745	0.5
C31	0.71688	0.48972	0.5

C32	0.69839	0.51389	0.5
C33	0.65957	0.5153	0.5
C34	0.52395	0.65157	0.5
C35	0.52508	0.69009	0.5
C36	0.5026	0.71235	0.5
C37	0.47852	0.69368	0.5
C38	0.4774	0.65485	0.5
C39	0.47556	0.33368	0.5
C40	0.47675	0.29484	0.5
C41	0.50074	0.27619	0.5
C42	0.52299	0.29848	0.5
C43	0.5218	0.33698	0.5
C44	0.34169	0.51979	0.5
C45	0.30319	0.52121	0.5
C46	0.28076	0.49883	0.5
C47	0.29925	0.47454	0.5
C48	0.33807	0.47312	0.5
N49	0.50362	0.23615	0.5
C50	0.48977	0.16926	0.5
C51	0.48589	0.21083	0.5
N52	0.50533	0.7524	0.5
C53	0.48734	0.77764	0.5
C54	0.49098	0.81924	0.5
N55	0.75691	0.48669	0.5
C56	0.78227	0.50449	0.5
C57	0.82384	0.50054	0.5
C58	0.51438	0.83754	0.5
C59	0.51591	0.87636	0.5
C60	0.49423	0.8989	0.5
C61	0.47105	0.88045	0.5
C62	0.46952	0.84194	0.5
C63	0.84197	0.47699	0.5
C64	0.88077	0.47516	0.5
C65	0.90347	0.49668	0.5
C66	0.8852	0.52002	0.5
C67	0.8467	0.52184	0.5
C68	0.51322	0.15111	0.5

C69	0.51501	0.1123	0.5
C70	0.49355	0.08962	0.5
C71	0.47032	0.10791	0.5
C72	0.46853	0.14641	0.5
C73	0.4949	0.04848	0.5
C74	0.49529	0.94004	0.5
C75	0.94461	0.49523	0.5
C76	0.9786	0.49456	0.5
C77	0.49553	0.97404	0.5
C78	0.49539	0.01448	0.5
N79	0.24073	0.50186	0.5
C80	0.21537	0.484	0.5
C81	0.1738	0.48797	0.5
C82	0.15567	0.51156	0.5
C83	0.11686	0.5134	0.5
C84	0.09417	0.49184	0.5
C85	0.11244	0.46845	0.5
C86	0.15094	0.46662	0.5
C87	0.05303	0.49329	0.5
C88	0.01904	0.49396	0.5
H89	0.42271	0.61933	0.5
H90	0.37518	0.57253	0.5
H91	0.62314	0.56858	0.5
H92	0.57528	0.61719	0.5
H93	0.51533	0.51519	0.5
H94	0.57488	0.36935	0.5
H95	0.62243	0.41606	0.5
H96	0.37447	0.41987	0.5
H97	0.42231	0.37118	0.5
H98	0.48225	0.47321	0.5
H99	0.64465	0.44019	0.5
H100	0.70887	0.44022	0.5
H101	0.71022	0.54234	0.5
H102	0.65158	0.54506	0.5
H103	0.55269	0.64046	0.5
H104	0.5522	0.70471	0.5
H105	0.45	0.70533	0.5

H106	0.4477	0.64663	0.5
H107	0.44584	0.34181	0.5
H108	0.45056	0.27862	0.5
H109	0.55276	0.29052	0.5
H110	0.55057	0.34801	0.5
H111	0.35306	0.54843	0.5
H112	0.28877	0.54844	0.5
H113	0.28735	0.44612	0.5
H114	0.34613	0.44338	0.5
H115	0.45519	0.21352	0.5
H116	0.45667	0.77469	0.5
H117	0.77955	0.53518	0.5
H118	0.54043	0.82109	0.5
H119	0.54318	0.8907	0.5
H120	0.44238	0.89174	0.5
H121	0.44225	0.82761	0.5
H122	0.82532	0.45107	0.5
H123	0.8949	0.44777	0.5
H124	0.89671	0.5486	0.5
H125	0.83258	0.54923	0.5
H126	0.53916	0.16773	0.5
H127	0.54238	0.09814	0.5
H128	0.44171	0.09647	0.5
H129	0.44117	0.16056	0.5
H130	0.21814	0.45332	0.5
H131	0.17232	0.53748	0.5
H132	0.10274	0.54079	0.5
H133	0.1009	0.43988	0.5
H134	0.16506	0.43924	0.5

Table S2. Comparison of ORR activities with other catalysts.

Samples	Half-wave potential (V *vs.* RHE)	Onset potential (V *vs.* RHE)	Ref.
TAPP-Co-COF	0.66	0.80	This work
JUC-606	0.7	0.79	Chemical Research in Chinese Universities, 2022, 38, 167-172.
Azo-COF	0.68	0.88	Angew. Chem. Int. Ed.; 2022, 134, e202209583.
JUC-528	0.7	0.83	J. Am. Chem. Soc. 2020, 142, 8104-8108.
Co-TP-COF	0.73	0.81	Chem Com. 2021, 57, 12619-12622.
Se/NC	0.77	0.88	Angew. Chem. Int. Ed 2023, 62, e202219191.
FeNi-CoP-800	0.80	0.96	Appl. Catal. B Environ. 2019, 243, 204-211.
Co_3HITP_2	0.8	0.91	Angew. Chem. Int. Ed. 2020, 59, 286-294.
Co-TPP/CNT	0.81	0.86	J. Energy Chem. 2021, 53, 77-81.
Co-TAPP-COF-Fe	0.84	0.95	Chem.- Asian J. 2020, 15, 1963-1969.
CoCOF-Py-0.05rGo	0.765	0.84	Dalton Trans. 2017, 46, 9344-9348.
CoTAPP-BDTA-COF	0.8	0.93	Angew. Chem. Int. Ed.; 2022, 61, e202213522.
